# Yeast diversity in relation to the production of fuels and chemicals

**DOI:** 10.1038/s41598-017-14641-0

**Published:** 2017-10-27

**Authors:** Jia Wu, Adam Elliston, Gwenaelle Le Gall, Ian J. Colquhoun, Samuel R. A. Collins, Jo Dicks, Ian N. Roberts, Keith W. Waldron

**Affiliations:** 1 The Biorefinery Centre, Quadram Institute Bioscience, Norwich Research Park, Colney, Norwich, NR4 7UA UK; 2 The Analytical Sciences Unit, Quadram Institute Bioscience, Norwich Research Park, Colney, Norwich, NR4 7UA UK; 3 The National Collection of Yeast Cultures, Quadram Institute Bioscience, Norwich Research Park, Colney, Norwich, NR4 7UA UK

## Abstract

In addition to ethanol, yeasts have the potential to produce many other industrially-relevant chemicals from numerous different carbon sources. However there remains a paucity of information about overall capability across the yeast family tree. Here, 11 diverse species of yeasts with genetic backgrounds representative of different branches of the family tree were investigated. They were compared for their abilities to grow on a range of sugar carbon sources, to produce potential platform chemicals from such substrates and to ferment hydrothermally pretreated rice straw under simultaneous saccharification and fermentation conditions. The yeasts differed considerably in their metabolic capabilities and production of ethanol. A number could produce significant amounts of ethyl acetate, arabinitol, glycerol and acetate in addition to ethanol, including from hitherto unreported carbon sources. They also demonstrated widely differing efficiencies in the fermentation of sugars derived from pre-treated rice straw biomass and differential sensitivities to fermentation inhibitors. A new catabolic property of *Rhodotorula mucilaginosa* (NCYC 65) was discovered in which sugar substrate is cleaved but the products are not metabolised. We propose that engineering this and some of the other properties discovered in this study and transferring such properties to conventional industrial yeast strains could greatly expand their biotechnological utility.

## Introduction

Large amounts of fossil-derived energy and chemicals are consumed globally each year. Unfortunately, the fossil resources, which are responsible for about 75% of the anthropogenic emission of CO_2,_ still account for more than 80% of our energy and 90% of our organic chemical needs^1–3^. Alternative energy sources are therefore of considerable interest and have been widely investigated, including sources such as water, solar, wind, nuclear fission/fusion and biomass^4^. As a possible alternative technology to petroleum refining, bio-refining shows many advantages with the potential of improving the quality of soil, water and air^5^. Ideally, bio-refining processes are needed to convert different feedstocks including biomass from municipal waste, agricultural waste, plant residues and industrial wastes to fuels or chemicals^6^. A priority list of chemicals which might be derived from biomass has been compiled by the US Department of Energy (DOE), including key renewable chemical targets such as ethanol, arabinitol, succinic acid, lactic acid and levulinic acid^4,7^.

It is the microorganism employed which determines the category of products produced from such substrates. Yeasts are fungi capable of converting sugars to a range of metabolites. They are widely used in the food industry for producing bread and wine^8–10^. *Saccharomyces cerevisiae* has been considered as the preferred yeast for ethanol fermentation, and can grow on simple sugars such as glucose, and on the disaccharide sucrose. However, more species of yeasts have been reported as being able to produce not only ethanol but also some highly-sought-after chemicals^11^. Therefore, diverse yeasts might have the potential to produce a range of bio-products via fermentation of different substrates.

In this study, to further explore the untapped potential of yeasts, and to inform future yeast screening studies, we have investigated and compared a specially selected set of genetically highly diverse yeast strains for their natural comparative abilities to ferment a range of carbon sources and to produce metabolites of potential interest in the renewable chemicals industry. Moreover, we have assessed how those selected yeast strains react to complex conditions when converting glucose from pretreated raw material to elucidate the potential of such yeast strains in industrial bio-refining.

## Results

### Growth of diverse yeasts on different carbon sources and their fermentation products

**Figure 1:**
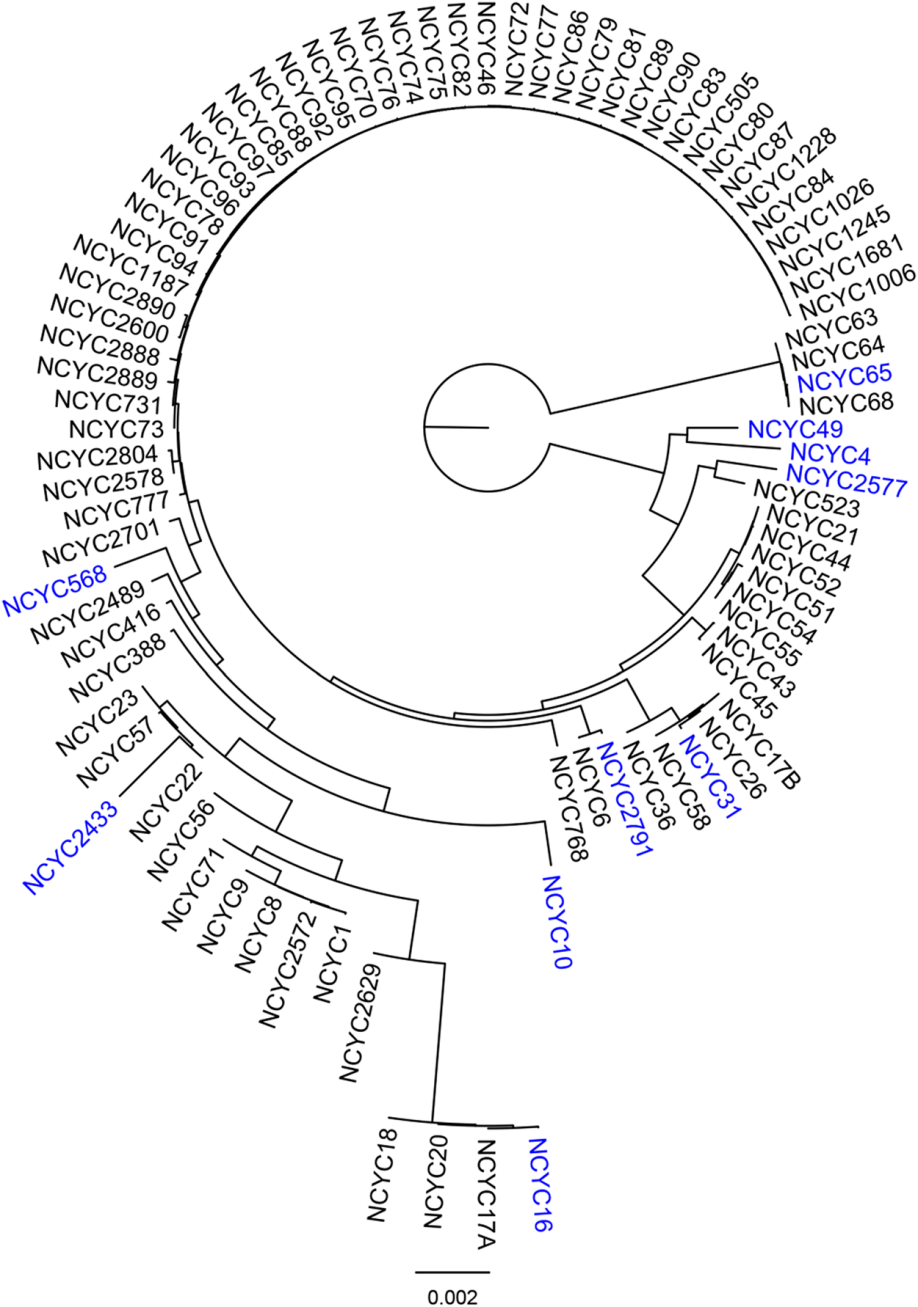
Diverse yeast panel. A phylogenetic tree was estimated from 94 genome contig files. The resulting Newick tree file was input to the Core Collection of Diverse taxa (CCD) software^48^ and the 10 maximally diverse yeast strains were selected under the Phylogenetic Diversity (PD) principle (shown in blue font). For clarity, 8 taxa with very long branches are not included in the figure.

The 11 yeast strains selected for this study (Fig. 1 and Supplementary Table S1) were chosen to present the maximum span of phylogenetic variation contained within the National Collection of Yeast Cultures (NCYC) collection, one of the world’s largest yeast strain collections comprising approximately 4,000 diverse strains. The strain set comprises isolates from both the Ascomycota and Basidiomycota phyla, which are thought to have diverged over 400 million years, with strains that are derived from different environmental conditions and which thrive on a range of carbon sources. Additional studies suggest (data not shown) this taxonomic diversity is also mirrored by considerable between-strain genetic, genomic and phenotypic diversity, making it highly valuable as an exemplar dataset for biotechnological analysis.

**Figure 2:**
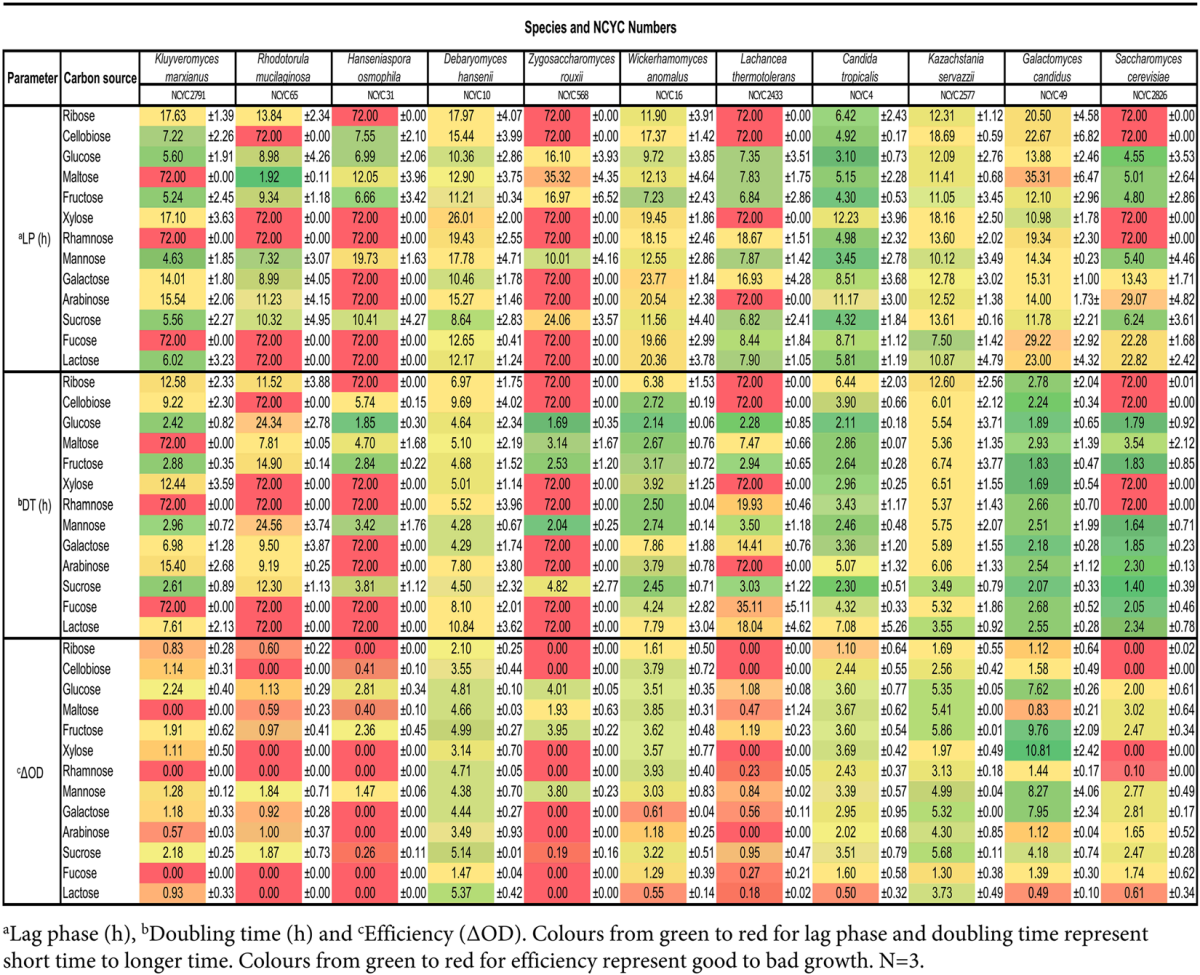
Growth of 11 yeast strains on 13 selected sugars comprising Lag phase (h), Doubling time (h) and Efficiency (ΔOD). Colours from green to red for lag phase and doubling time represent short time to longer time. Colours from green to red for efficiency represent good to bad growth. This experiment was performed in triplicates and raw data was processed by using PRECOG software.

Initially, aerobic growth of the 11 diverse yeasts on 13 different sugars (including pentose (C5), hexose (C6) and disaccharides (DIS) derived from plant tissues and microbial fermentation products) were tracked for 72 hours by recording turbidity every 30 mins. Subsequently, the lag phase (LP), doubling time (DT) and efficiency (ΔOD) were calculated by using PRECOG (see  Methodology). Figure 2 employs a red-amber-green colour coding to show the extensive variation in LP, DT and ΔOD. The way these values reflect properties of growth curves are described in Supplementary Figure S1. For LP and DT, darker green reflects a shorter time taken, red indicates a longer time taken. For ΔOD, darker green indicates stronger growth and red means weaker growth. It is apparent that there is considerable diversity in the LP, DT and ΔOD between the different strains. Only NCYC 2577 and NCYC 10 could grow on all 13 sugars. NCYC 49, NCYC4 and NCYC 16 grew on fewer sugars and those strains also exhibited generally shorter doubling times or lag phases. Similarly, NCYC 2791, NCYC 65 and NCYC 2826 could not grow on either rhamnose or fucose. Maltose was not an ideal carbon source for NCYC 2791. Cellobiose, xylose and lactose were not utilised effectively by NCYC 65 and ribose, cellobiose and xylose were poorly utilised by NCYC 2826. NCYC 2433 gave a short LP and DT on maltose, galactose and mannose but the growth (ΔOD) was poor. NCYC 31 and NCYC 568 could both grow efficiently on glucose, fructose and mannose, but poorly on sucrose. NCYC 568 grew better than NCYC 31 on maltose whilst NCYC 31 grew better than NCYC 568 in cellobiose. These results illustrated the genetic diversity of yeast strains that significantly differed in both sugar utilization and growth.

**Figure 3:**
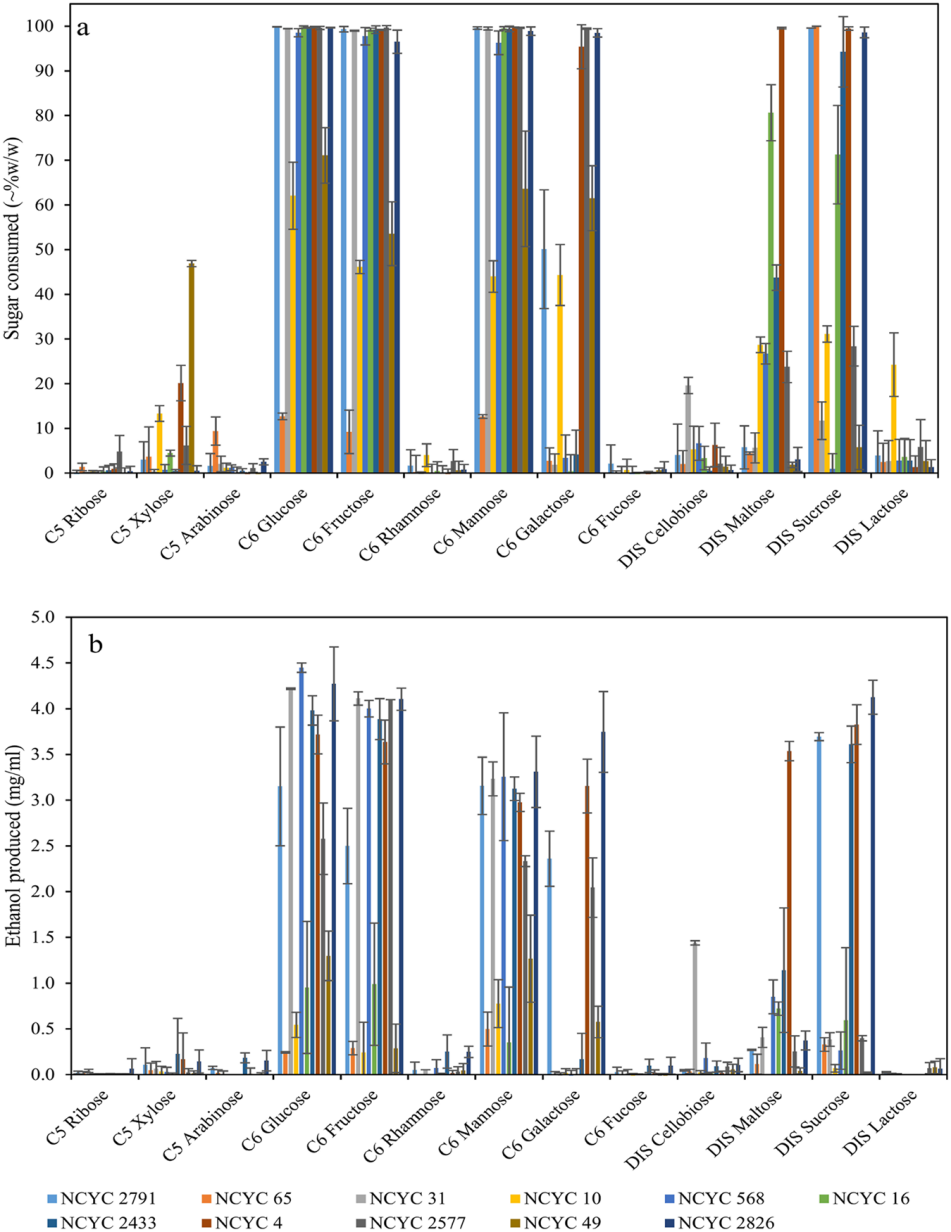
Percentage of sugar consumed by 11 selected yeast strains and the yield of ethanol in different samples. Figure 3a shows the percentage consumption of different sugars used by selected yeast strains. Figure 3b shows the ethanol yield from yeast fermentation on different sugars. Concentration of given sugars was 10 mg/ml individually. Therefore, theoretical maximum ethanol yield was up to 5.11 mg/ml. n = 3.

Final fermentation liquors of the (anaerobic) yeast fermentations were analysed for the levels of unmetabolized sugar substrates (Fig. 3a) and ethanol production (Fig. 3b) by HPLC. Less than 5% of ribose, rhamnose, arabinose and fucose were consumed by any strain. In contrast, the other sugars were generally completely consumed by at least one or more strains. Therefore, ribose, rhamnose, arabinose and fucose were not investigated further. Nearly all the glucose, fructose and mannose were consumed by most strains (along with the substantial production of ethanol) except NCYC 65, NCYC 10 and NCYC 49. A smaller number of strains quantitatively consumed and fermented galactose and sucrose to ethanol. Xylose was generally poorly consumed, although NCYC 49 utilised nearly 50%. However, none of the xylose fermenters produced significant amounts of ethanol.

Interestingly, NCYC 16 consumed many of the sugars to a large degree but it failed to produce the expected stoichiometric levels of ethanol. Whilst NCYC 16 consumed all the glucose, it produced only half the amount of ethanol produced by NCYC 568. Surprisingly, NCYC 65 consumed virtually all sucrose whilst producing almost no ethanol. This wide range of fermentation behaviours indicated that metabolic products other than ethanol might be produced.

### Analysis of Metabolites produced by the 11 selected NCYC yeast strains grown on different sugars

**Figure 4:**
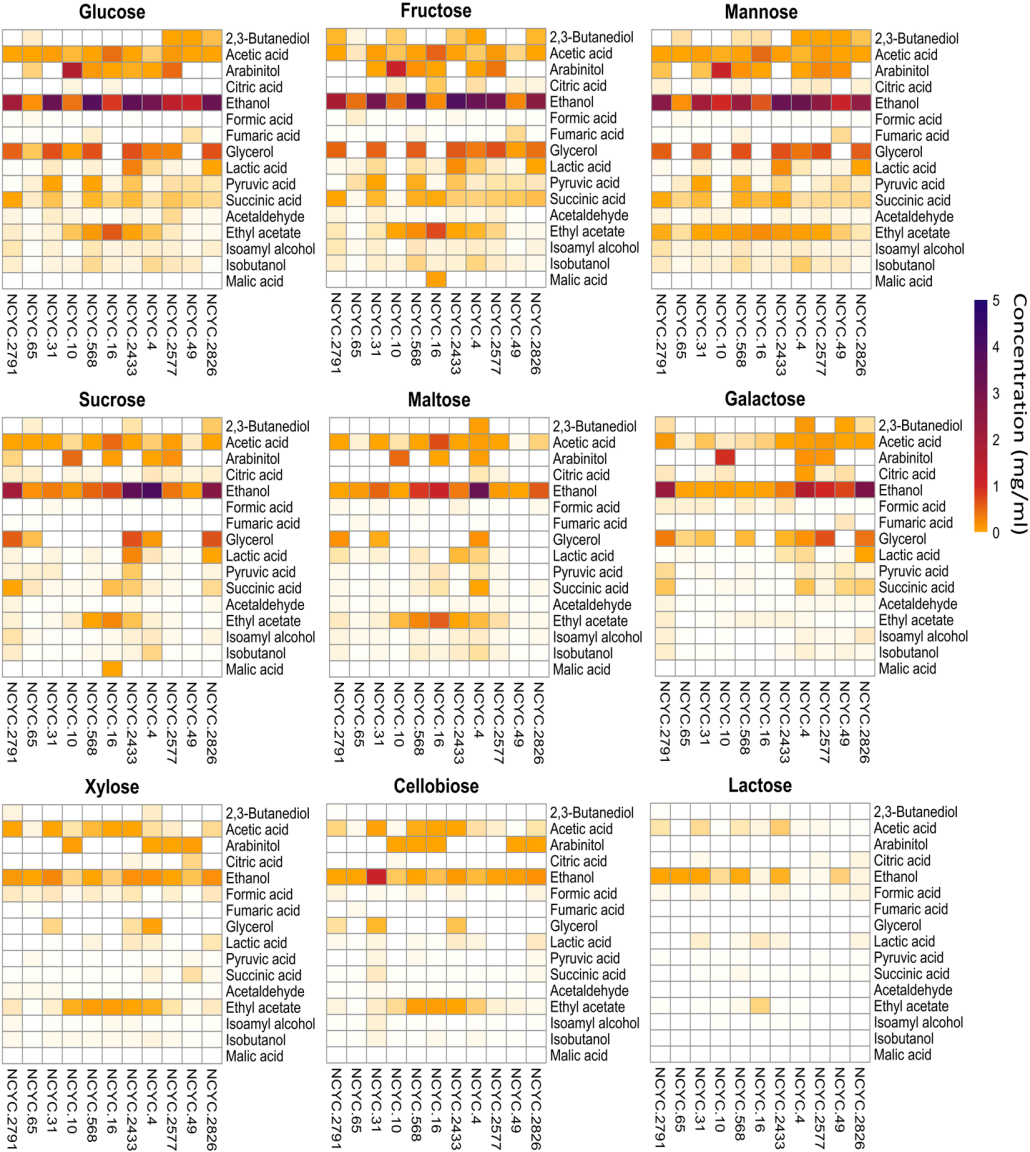
Heatmaps of quantities of 16 chemicals produced by 11 diverse yeast strains from 9 sugars. Concentration of chemicals was calculated as w (mg) per v (ml) of fermented liquor. The changes of concentration are represented as colour changing from white (0 mg/ml) to dark purple (5 mg/ml). Data was selected from 4 replicates and then processed by using R.

To assess the production of other metabolites which could be of industrial significance, fermented liquors were analysed by a new high throughput nuclear magnetic resonance (NMR) method. This enabled the identification and quantification of 16 significant metabolites. Data was collected and is presented in Fig. 4. Each graph shows 16 chemicals produced by 11 strains from one of 9 sugars (ribose, rhamnose, arabinose and fucose had been eliminated from the study). Concentrations of chemicals were calculated as mg/ml of fermented liquor and used to conditionally format Fig. 4 with colour coding from white (compounds yield from 0 mg/ml to 0.002 mg/ml which could not be confidently distinguished from baseline noise) through orange and red to Navy (5 mg/ml) (see Methodology). Considerable variation was observed in the profile of chemical products produced by the different strains as affected by the sugar substrates, extending considerably the information present in the UK NCYC, CRC and other databases.

Of the 16 chemicals quantified, 2,3-butanediol, acetic acid, arabinitol, citric acid, ethanol, glycerol, lactic acid, pyruvic acid, succinic acid, ethyl acetate and malic acid were produced in the highest quantities. The other metabolites were produced in trace quantities. Ethanol was the most common product detected after fermentation of all sugars as well as acetic acid. Notwithstanding differences between the yeasts, arabinitol, glycerol, succinic acid and ethyl acetate were produced from 8 sugars but not from lactose. Although the yield of lactic acid was generally low; it was still produced from all sugars. Citric acid was produced widely but negligibly from most of sugars but significantly from galactose. Like citric acid, 2,3-butanediol was significantly produced by only maltose and galactose. Higher yields of pyruvic acid were detected from glucose, fructose, mannose and sucrose compared with the other sugars. Interestingly, malic acid was only produced by NCYC 16 and then only from fructose. Ethyl acetate was significantly produced from 8 of 9 sugars except galactose by several strains. NCYC 16 produced highest amount of ethyl acetate from those sugars. Arabinitol was also significantly produced from 8 of the 9 sugars (excluding lactose) by several strains especially by NCYC 10.

Multiple additional products were also significantly detected by NCYC 568 and NCYC 2577 fermentation from several fermented sugars. For example, NCYC 568 could produce glycerol and ethyl acetate in addition to ethanol from several sugars. Similarly, 3 different readily quantifiable chemicals could be produced by NCYC 2577 - in addition to ethanol: acetic acid was produced from glucose, fructose, mannose, sucrose and galactose; glycerol was produced from glucose, fructose, mannose and galactose. Interestingly, ethanol was insignificantly produced from cellobiose by all strains except NCYC 31.

**Figure 5:**
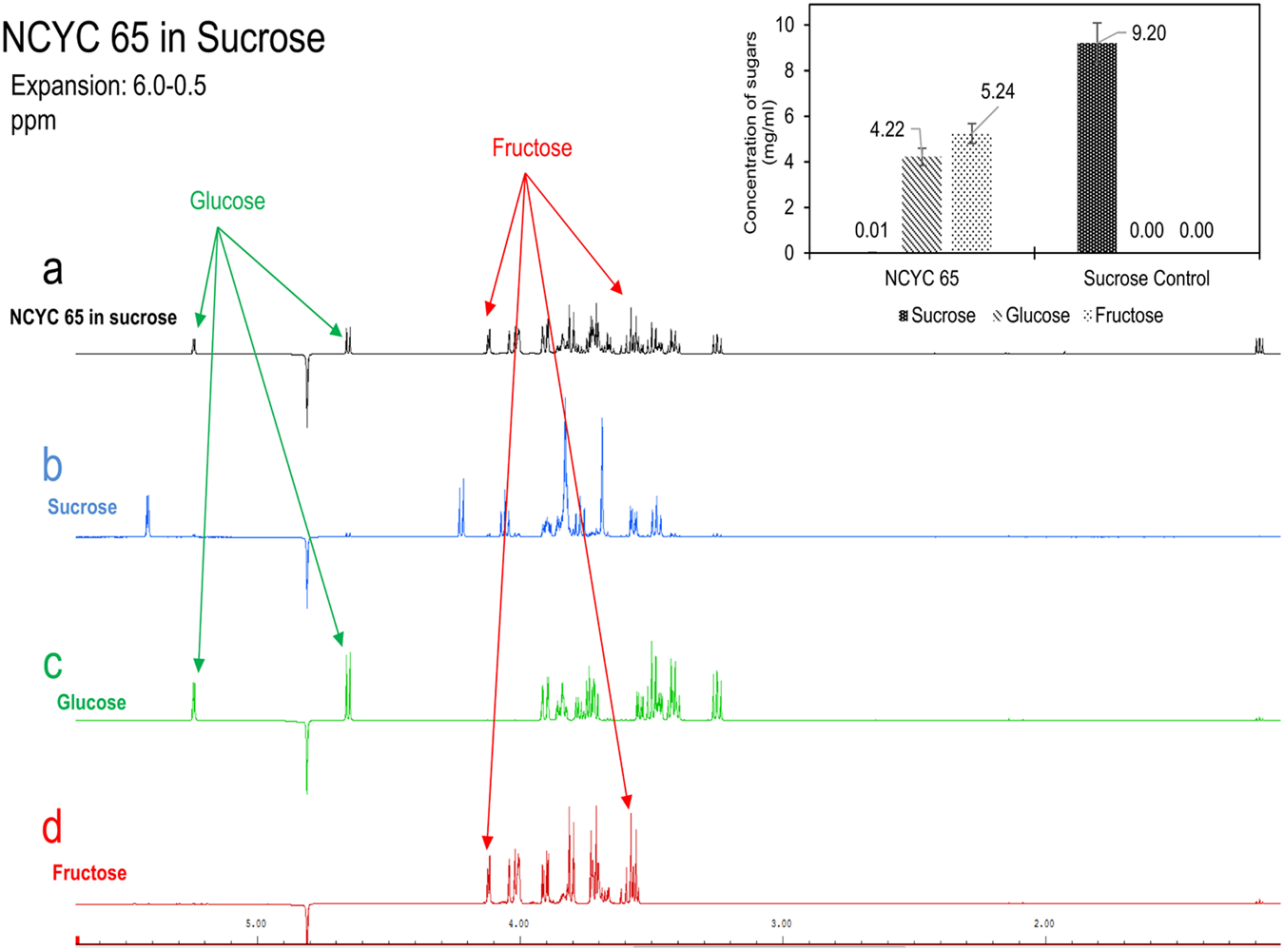
The NMR spectrum of NCYC 65 in sucrose (**a**), sucrose standard (**b**), glucose standard (**c**) and fructose standard (**d**). Spectrum (**a**) contains the same peaks as spectrum (**c**) and spectrum (**d**) which means that glucose and fructose are present in spectrum (**a**). No similarity was identified between spectrum (**a**) and spectrum (**b**) which means no sucrose in spectrum (**a**). The graph on the top right shows the quantification of glucose, fructose and sucrose of sample “NCYC 65 in sucrose” and sample “sucrose control”. This illustrates sucrose was completely degraded into glucose and fructose by yeast strain NCYC 65. Results were collected from triplicates.

NCYC 65, the strain set’s single *Basidiomycetous* yeast, consumed nearly 100% of sucrose without producing a reasonable amount of any product (Figs 3 and 4). This observation was investigated further (Fig. 5). Closer examination of the NMR spectra showed that the fermentation liquor from NCYC 65 after growth on sucrose showed a loss of peaks associated with sucrose (Fig. 5a and b), but showed instead peaks associated with glucose (Fig. 5c) and fructose (Fig. 5d). Hence, sucrose was simply cleaved into glucose and fructose, and NCYC 65 consumed neither glucose nor fructose. NCYC 65 could therefore potentially provide a direct biological route to industrial invertase function without necessarily purifying the enzyme(s).

### Simultaneous saccharification and fermentation (SSF) of diverse set of yeasts on hydrothermally pre-treated rice straw

**Figure 6:**
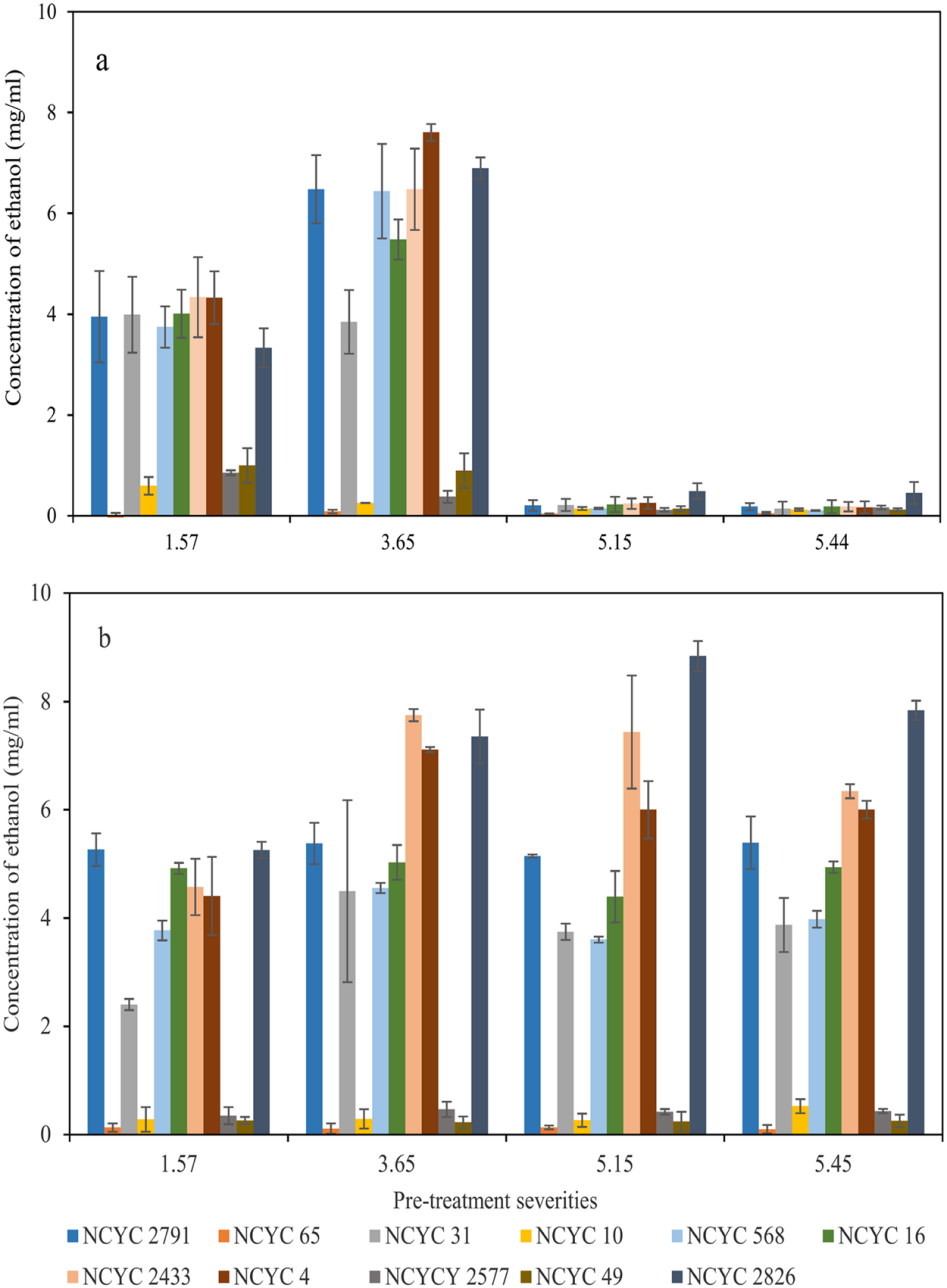
Ethanol yields of fermentation of 11 yeasts on hydrothermally pre-treated rice straw. Rice Straw had been pre-treated with 4 different severities. Figure 6(a) shows the concentration of ethanol produced from pre-treated rice straws which contained the pre-treatment liquor. Figure 6(b) shows the concentration of ethanol produced from hydrothermally pre-treated, then washed rice straw. Each coloured bar shows the concentration of ethanol produced by individual yeast strains. Concentration of substrate in fermentation slurry was 5% and maximum theoretical ethanol yield is 9.89 mg/ml. Concentration of ethanol was calculated as mg per ml of fermented liquor (w/v). N = 2.

The fermentation conditions created using pre-treated raw materials are much more complex than those involving purified sugars. To evaluate the ability of the different yeast strains to utilise real world biomass substrates to produce ethanol, rice straw material characterised previously by Wood, *et al*.^12^ was hydrothermally pre-treated at severities 1.57, 3.65, 5.15 and 5.44 before SSF for 72 h (pre-treatment severity of rice straw see  Methodology). At an initial substrate loading of 5% w/w (50 mg/ml, see  Methodology), the maximum theoretical ethanol yield is 9.89 mg/ml (50 mg/ml raw rice straw containing 38.66% glucose potentially gives a glucose concentration of 19.33 mg/ml. Thus, according to the equation: C_6_H_12_O_6_ (glucose) = 2CH_3_CH_2_OH (ethanol) + 2CO_2,_ maximum ethanol theoretically produced should be no more than 9.885 mg/ml). Initially, SSF was carried out using the complete pre-treated slurry. The results (Fig. 6a) show that ethanol was produced from rice straw that had been pre-treated up to a severity of 3.65. At the higher severities (5.15 and 5.45), virtually no ethanol was produced. This was assumed to be due to the retention of fermentation inhibitors in the slurry as described by Wood, *et al*.^12^. These are derived from the degradation of some pentoses and hexoses to furfural (2-FA), 5-hydroxymethylfurfural (5-HMF), formic acid and levulinic acid.

Therefore, pre-treated rice straw samples were washed with distilled water 3 times prior to resuspension and evaluation for SSF. The results are shown in Fig. 6b and demonstrate that the concentration of ethanol increased for the samples pre-treated at a severity of 5.15 and dropped a little at a severity of 5.45. Most strains could produce ethanol by converting the sugars released from the cell wall of rice straw. Consistent with the results shown in Fig. 3, NCYC 65, NCYC 10 and NCYC 49 could not convert sugars to ethanol efficiently. In contrast, NCYC 2577 which was shown as an ethanol producer in Fig. 3, could not produce ethanol significantly from any of the pre-treated samples indicating a very significant susceptibility to fermentation inhibitors.

## Discussion

In this study, the paucity of xylose and arabinose fermenters is notable. There is considerable interest in biofuel production from lignocellulosic hemicelluloses^13^ since xylose is the second most abundant fermentable sugar in lignocellulosic materials. Naturally-occurring pentose-fermenting yeasts are limited, and include *Pichia* (*Scheffersomyces stipites*)^14^; *Candida shehatae* ^13,15^. Hence there has been significant effort to genetically modify yeasts to enhance this property whilst improving hydrolysate inhibitor tolerance^16^. Pentoses and lactose can be fermented by bacteria, fungi and certain yeast strains^17–19^.

Ethyl acetate is an important platform chemical that can be used as a microbiologically-degradable and environmentally friendly solvent in the manufacture of food, glues, inks and perfumes. The world’s annual demand of ethyl acetate was 2.5 million tons^20^, however recent production of it was only 1.7 million tons mainly produced via chemical processes^21^. In this study, ethyl acetate was produced in largest quantities by NCYC 16 (*Wickerhamomyces anomala*) from a range of sugar substrates, extending the findings of Walker^22^ who identified *Pichia anomala* (*recently renamed as Wickerhamomyces anomala*) as a high ethyl acetate producer and compared it with *S*. *cerevisiae*, and Kurita^23^ and Rojas, *et al*.^24^ who had carried out fermentation of *P*. *anomala* on malt agar medium and glucose respectively. The present study has further demonstrated that the yield of ethyl acetate varies significantly when fermented in different sugars (glucose, mannose, sucrose and maltose). For example, ethyl acetate was produced more from fructose than from glucose by NCYC 16. This may be due to the efficiency of conversion via different metabolic pathways of *P*. *anomala*. Glucose is firstly metabolised via multiple pathways - to glucose-6-phosphate (glucose-6-P) and then portioned for growing biomass (cells) or converted to fructose-6-P (fructose-6-phosphate)^25,26^. In contrast, fructose is directly metabolised to fructose-6-P and then further irreversibly phosphorylated to fructose-1, 6-bisphosphate^27^. Yields may also be affected by levels of oxygen which can inhibit or enhance ethyl acetate production^26,28,29^. Further research will be required to optimise the conditions for enhancing ethyl acetate production by NCYC 16 and to compare its phenotype to additional closely related NCYC strains. Preliminary results have strongly-indicated that ethyl acetate production may be significantly enhanced by intermediate levels of air or oxygen (results not shown).

Arabinitol is currently synthesised via a chemical reaction requiring catalysis at high temperature, and it is an interesting platform chemical that has the potential to be used as a non-nutritive sweetener and as a feedstock in producing ethylene glycol, propylene, enantiopure compounds, arabinoic and xylonic acids^30–32^. This study also observed, we believe for the first time, that high arabinitol yields could be produced by NCYC 568 (*Zygosaccharomyces rouxii*) and NCYC 2577 (*Kazachstania servazzii*) from a range of sugars (both hexoses and pentoses), and in NCYC49 (*Galactomyces candidus*) from mannose. NCYC 10 (*Debaryomyces hansenii*) was also found to produce considerable quantities of arabinitol at levels higher even than ethanol. This confirms and extends previous studies that reported accumulation of arabinitol from a range of carbon sources^33,34^.

NCYC 31 was the only strain in the set which could convert cellobiose largely into ethanol due to its ability to produce β-glucosidase^35^ and may provide useful genetic information for bioethanol production. Similarly, NCYC 65 may be a potential enzyme producer which can simply cleave sucrose to glucose and fructose monomers, presumably by invertase activity. However, how NCYC 65 obtains energy is not clear – a careful mass balance will need to be carried out to clarify whether any of the glucose or fructose are metabolised. It is interesting that NCYC 65 grows more rapidly in sucrose than in glucose, although we did not assess growth of the yeast in both fructose and glucose mixtures. An understanding of the genetics and biochemistry in comparison with other strains might be used as a first step to improve conversion of sucrose to glucose and fructose. NCYC 65 was unable to ferment any of the 13 sugars significantly. However, Lee *et al*. reported that a strain of *R*. *mucilaginosa* could produce acetylxylan esterase^36^. Such an esterase could remove certain substituents of hemicelluloses such as acetyl groups, which could potentially enhance the activity of xylanases^36–38^. Moreover, a high fatty acids accumulation strain of *R*. *mucilaginosa* was reported in the research of Li, *et al*.^39^. They mentioned those fatty acids could be used for biodiesel.

The fermentation conditions created using pre-treated raw materials will be much more complex than environments created by purified sugars. In this study, each strain’s ability to produce ethanol was assessed and compared between enzymatic hydrolysates of rice straw and fermentation on purified sugars. Results were very consistent between the two substrates for all strains except NCYC 2577. NCYC 2577 could ferment purified glucose but was not very effective at fermenting the glucose from rice straw. It is possible that NCYC 2577 is particularly sensitive to inhibitors at very low levels (that might remain even after washing). Inhibitors of yeast fermentation are produced during unavoidable pre-treatments and yeasts can demonstrate considerable variation in their response to such chemicals^40^. There are some ways to reduce the effects of inhibitors and enhance the yield of products. For example, detoxification of hydrolysates and the use of fully or partially inhibitor resistant yeast strains^41–43^. It may be possible to improve the resistance of yeasts to inhibitors by genetic improvement^40^. Additional fine-tuning of the pre-treatment process may also be helpful^44^.

In conclusion, this study presented information on yeast behaviours when grown on a range of pure carbon sources, identifying the key metabolites produced by some of strains across the different sugar substrates. It has demonstrated that the chosen phylogenetic diversity of the strain set was matched by phenotype diversity, highlighting the importance of screening widely across the vast yeast taxonomy for key bio-industrial traits. Furthermore, by evaluating the ability of yeast strains to ferment rice straw hydrolysates and comparing those data with fermentation of purified sugars, this study has highlighted the challenges that need to be addressed when attempting to exploit yeasts industrially. Nevertheless, we have identified significant gaps in knowledge that would be worthy of further research, including the possibility of creating ethyl acetate or D-arabinitol from raw materials, and the exploitation of NCYC 65 for sucrose hydrolysis.

## Methodology

### Yeast strains and carbohydrates

The genomes of a set of 94 diverse yeast strains selected from the National Collection of Yeast Cultures (NCYC) were paired-end sequenced at the Earlham Institute, Norwich (formerly the Genome Analysis Centre) using an Illumina HiSeq. 2000 sequencer and TruSeq libraries, with 2 × 100 bp reads derived from inserts of size 500 bp. Raw reads for a subset of these strains may be found at the NCYC OpenData website (http://opendata.ifr.ac.uk/NCYC/)^45^. Draft genome assemblies were obtained from the raw reads for each of the 94 strains using the A5^46^ assembly pipeline (20130326 version). A phylogenetic tree was estimated from the resulting 94 genome contig files using the FFP software^47^ with the RY alphabet and a word size of k = 14. The resulting Newick tree file was input to the Core Collection of Diverse taxa (CCD) software^48^ and the 10 maximally diverse yeast strains were selected under the Phylogenetic Diversity (PD) principle (Fig. 1, Supplementary Table S1). These 10 strains were chosen for further experimentation, in addition to NCYC 2826, a *Saccharomyces cerevisiae* strain frequently used by us as a cross-experiment standard. Yeasts were firstly transferred from glycerol stocks into agar plates and further grown in yeast nitrogen base (YNB) +1% glucose (Formedium, Hunstanton, Norfolk, United Kingdom) at 25 °C for 72 hours before use. The 13 laboratory-purified sugars were obtained from (Sigma-Aldrich, Gillingham, Dorset, United Kingdom). 500 ml of a 10 mg/ml solution of each sugar in YNB was prepared and autoclaved.

### Growth of selected yeast strains in 13 sugars

All samples were prepared in triplicate. 20 µl of each selected yeast strain was transferred into a 96 well reader plates (Thermo Fisher Scientific, Waltham, MA USA) which contained 180 µl of each sugar (10 mg/ml). The reader plate was placed in VersaMax ELISA Microplate Reader (Molecular Devices, Sunnyvale, CA USA) and incubated at 25 °C for 72 hours by which time growth had ceased. Growth of yeasts was recorded every 30 min during the 72-hour incubation and then further processed by using PRECOG^49^ to obtain parameters shown in Fig. 2.

### Small-scale fermentation on 13 sugars

Small-scale fermentation was carried out in triplicate in 96 deep well (2 ml) plates (Geriner Bio-One Ltd, Brunel Way, UK). 980 µl of sugar solution (10 mg/ml) was added in to a set of 11 wells and then one of the 11 yeasts (20 µl) was added to each well. This was repeated for all sugars. Plates were sealed with a clear polypropylene PCR seal (STARLAB international GmbH, 22143 Hamburg, Germany). They were then, incubated on an orbital shaker (135 rpm) in a 25 °C incubation room for 72 hours. The fermentation was terminated by heating at 100 °C for 10 mins. After cooling on ice, the supernatants were filtered (0.2 µm pore size, Polyvinylidene difluoride (PVDF) filters) and analysed for residual sugars and ethanol by High Performance Liquid Chromatography (HPLC) using a Series 200 LC instrument (Perkin Elmer, Seer Green, United Kingdom) equipped with both a refractive index detector and photodiode array detector. Separations were performed on a BIO-RAD Aminex® HPX-87H organic acid analysis column (300 × 7.8 mm; BIORAD Cat # 1250140), protected by a matching guard column, eluting with 0.004 mol/l H_2_SO_4_ mobile phase at a flow rate of 0.6 ml/min, column temperature 65 °C. Injection volume was 25 µl.

### Metabolome profiling using ^1^H NMR

^1^H NMR was used to identify and quantify key metabolites in the triplicate fermentation liquors. Samples were prepared by mixing 300 µl of liquor with 300 µl of NMR phosphate buffer (comprising 8.4 g NaH_2_PO_4_, 3.3 g K_2_HPO_4_, 17.2 mg of sodium 3-(Trimethylsilyl)-propionate-d4 (TSP) as a chemical shift and concentration reference, 40 mg sodium azide in 200 ml deuterium oxide, pH 6.4). 500 µl aliquots of the mixture were transferred into 5-mm NMR tubes for spectral acquisition. The ^1^H NMR spectra were recorded at 600 MHz on a Bruker Avance spectrometer (Bruker BioSpin GmbH, Rheinstetten, Germany) running Topspin 3.2 software and fitted with a cryoprobe. Each ^1^H NMR spectrum was acquired with 64 scans, a spectral width of 12300 Hz, an acquisition time of 2.7 s, and a relaxation delay of 3.0 s. The “noesygppr1d” pre-saturation sequence was used to suppress the residual water signal with a low-power selective irradiation at the water frequency during the recycle delay and a mixing time of 10 ms. Spectra were transformed with a 0.3-Hz line broadening, manually phased, baseline corrected, and referenced by setting the TSP methyl signal to 0 ppm. The metabolites were quantified using the software Chenomx NMR suite 7.6™. Metabolites were identified using information found in the literature, on the web (Human Metabolome Database, http://www.hmdb.ca/), in the Chenomx standards library, or by use of the 2D-NMR methods, correlation spectroscopy (COSY), heteronuclear single quantum coherence (HSQC), and heteronuclear multiple bond coherence (HMBC). Some additional spectra of standards were run in-house to supplement those available in the Chenomx library. Heat-maps as presented were edited by using R (package “pheat-map”, color combination: white, orange, red, navy. For the purpose of showing a broad range of concentrations, the color scale was enhanced by setting BAIS to 5.65 and length to 17000 (2000 for 0 mg/ml to 1 mg/ml; 15000 for 1 mg/ml to 5 mg/ml), hence yellow and light orange represented compounds were produced as trace quantities. (https://www.r-project.org/).

### Raw materials

As described previously^12^ rice straw was grown in a rice paddy field at the Ba Vi National Park, Hanoi, Vietnam. After mature straws were harvested in spring 2012, the biomass was further fumigated and air-dried under ambient conditions (approx. 34 °C, 84% RH) at the Agricultural Genetics Institute, Hanoi, Vietnam^12^. Cell wall polysaccharides were previously identified as containing rhamnose, fucose, arabinose, xylose, mannose, galactose and glucose. Rice straw used in this study contains 39% ± 0.65% of glucose, 23% ± 0.31% of xylose, 4% ± 0.14% of arabinose and 1.4% ± 0.04% of galactose. Only minor concentration of rhamnose, mannose and fucose were present.

### Freeze milling

Rice straw (<2 cm) was milled in a 6700EFM Freezer/Mill (Spex Sample Prep, Stanmore, United Kingdom). Samples were pre-frozen in liquid nitrogen for 10 mins. Then, freeze milled for five mins. These samples were then pre-treated for SSF (explained below).

### Pre-treatment of milled rice straw

Milled rice straw was pretreated by using a BIOTAGE® Initiator + reactor (Biotage AB, Uppsala, Sweden). 4 samples (each 0.75 mg) had been weighed into 20 ml microwave pressure tubes. Water (14.25 ml) was then added into each of those tubes to give a 5% (w/w) suspension. Capped tubes were treated at severities 1.57, 3.65, 5.15, 5.45 (pre-treatment severities were adapted from report of Jacquet, *et al*.^50^ as those severities were selected from different thermal degradation zones. Tubes were cooled with compressed air to ambient, and stored at −20 °C before further experimentation.

### Simultaneous saccharification and fermentations (SSF) of pre-treated rice straw and pre-treated washed rice straw

SSF was conducted in 1 ml Matrix tubes (Thermo Fisher Scientific, Waltham, MA USA). Experiments on each pre-treatment severity regime were performed in duplicate. The pre-treated samples were defrosted and then each agitated to provide a uniform suspension. Whilst mixing with small magnetic stirrer bars, 937.5 µl slurry was transferred into each Matrix tube. Cellic® CTec2 (Novozymes, Denmark) (12.5 µl, 144 FPU) and one of the pre-grown yeast strains (each 50 µl) were then added. The tubes were sealed by screw caps and set into 96-tube Matrix plates were then incubated on shaker (135 rpm) at 25 °C for 72 hours. After fermentation, those tubes were heated at 100 °C for 10 min, then cooled on ice and centrifuged (3000 rpm) for 10 min. 400 µl supernatants of each sample were filtered using a Pall filter plate 0.2 µm (Pall Corporation, World Headquarters, Washington USA) then multi-pipetted into a 96 well deep-well plate for HPLC analysis. Ethanol standards were made for quantifying ethanol produced by yeast fermentation.

SSF of pretreated and washed rice straw was carried out in the same way. The washing involved centrifugation to sediment the pretreated residue, removal of the supernatant by decanting, followed by resuspension of the pellet in distilled water to 15 ml. This was carried out 3 times.

### Data availability statement

All data generated or analysed during this study are included in this published article.

## Electronic supplementary material

Supplementary Information
