# Supplementary material for: Yeast diversity in relation to the production of fuels and chemicals
Source: Sci Rep. 2017 Oct 27;7:14259. doi: 10.1038/s41598-017-14641-0 (PMC5660169; doi:10.1038/s41598-017-14641-0)

Supplementary Information

Yeast diversity in relation to the production of fuels and chemicals.

Jia Wu[1]; Adam Elliston[1]; Gwenaelle Le Gall[2]; Ian J Colquhoun[2]; Samuel R A Collins[1]; Jo Dicks[3]; Ian N Roberts[3]; Keith W Waldron[1*]

[1] The Biorefinery Centre, Quadram Institute Bioscience, Norwich Research Park, Colney, Norwich NR4 7UA, UK

[2]The Analytical Sciences Unit, Quadram Institute Bioscience, Norwich Research Park, Colney, Norwich NR4 7UA, UK

[3] The National Collection of Yeast Cultures, Quadram Institute Bioscience, Norwich Research Park, Colney, Norwich NR4 7UA, UK

*Corresponding Author:

Keith W. Waldron

Quadram Institute Bioscience

Norwich Research Park,

Norwich NR4 7UA, UK

Phone: +44-1603-255385; Fax: +44-1603-507723

Email: keith.waldron@quadram.ac.uk

Supplementary Table S1: Information and history of selected yeasts

Yeast Number	Name	NCYC number	Habitat	Year deposited	NCYC URL	Other URL	
**1**	*Kluyveromyces marxianus*	NCYC 2791			https://catalogue.ncyc.co.uk/kluyveromyces-marxianus-2791	CBS:	http://www.westerdijkinstitute.nl/Collections/BioloMICS.aspx?TableKey=14682616000000011&Rec=383&Fields=All
**2**	*Rhodotorula mucilaginosa*	NCYC 65		1921	https://catalogue.ncyc.co.uk/rhodotorula-mucilaginosa-65	ATCC	https://www.lgcstandardsatcc.org/products/all/20129.aspx?geo_country=gb#generalinformation
**3**	*Hanseniaspora osmophila*	NCYC 31	Bark of tree	1920	https://catalogue.ncyc.co.uk/hanseniaspora-osmophila-31	CBS	http://www.westerdijkinstitute.nl/Collections/BioloMICS.aspx?Table=CBS%20strain%20database&Rec=29&Fields=All
**4**	*Debaryomyces hansenii*	NCYC 10		1925	https://catalogue.ncyc.co.uk/debaryomyces-hansenii-10		
**5**	*Zygosaccharomyces rouxii*	NCYC 568	Concentrated black grape must	1959	https://catalogue.ncyc.co.uk/zygosaccharomyces-rouxii-568	CBS	http://www.straininfo.net/strains/105346/browser
**6**	*Wickerhamomyces anomalus*	NCYC 16		1921	https://catalogue.ncyc.co.uk/wickerhamomyces-anomalus-16	ATCC	https://www.atcc.org/~/ps/36905.ashx
**7**	*Zygosaccharomyces thermotolerans*	NCYC 2433	Fermenting Mirabelle plum jam	1992	https://catalogue.ncyc.co.uk/lachancea-thermotolerans-2433	CBS	http://www.westerdijkinstitute.nl/Collections/BioloMICS.aspx?Table=CBS%20strain%20database&Rec=3018&Fields=All
**8**	*Candida tropicalis*	NCYC 4		1928	https://catalogue.ncyc.co.uk/candida-tropicalis-4	CBS	http://www.westerdijkinstitute.nl/Collections/BioloMICS.aspx?Table=CBS%20strain%20database&Rec=1203&Fields=All
**9**	*Kazachchstania servazzii*	NCYC 2577	Soil	1994	https://catalogue.ncyc.co.uk/kazachstania-servazzii-2577	CBS	http://www.westerdijkinstitute.nl/Collections/BioloMICS.aspx?Table=CBS%20strain%20database&Rec=1878&Fields=All
**10**	*Galactomyces candidus*	NCYC 49	Milk	1929	https://catalogue.ncyc.co.uk/galactomyces-candidus-49		
**11**	*Saccharomyces cerevisiae*	NCYC 2826	Grape must	1998	https://catalogue.ncyc.co.uk/saccharomyces-cerevisiae-2826	CECT	http://www.straininfo.net/strains/283117/browser

Supplementary Figure S1. Phases of yeast growth highlighting the Lag Phase (LP), Exponential Phase (EP) and Stationary Phase, (SP) and the definition of Efficiency (Δ OD).

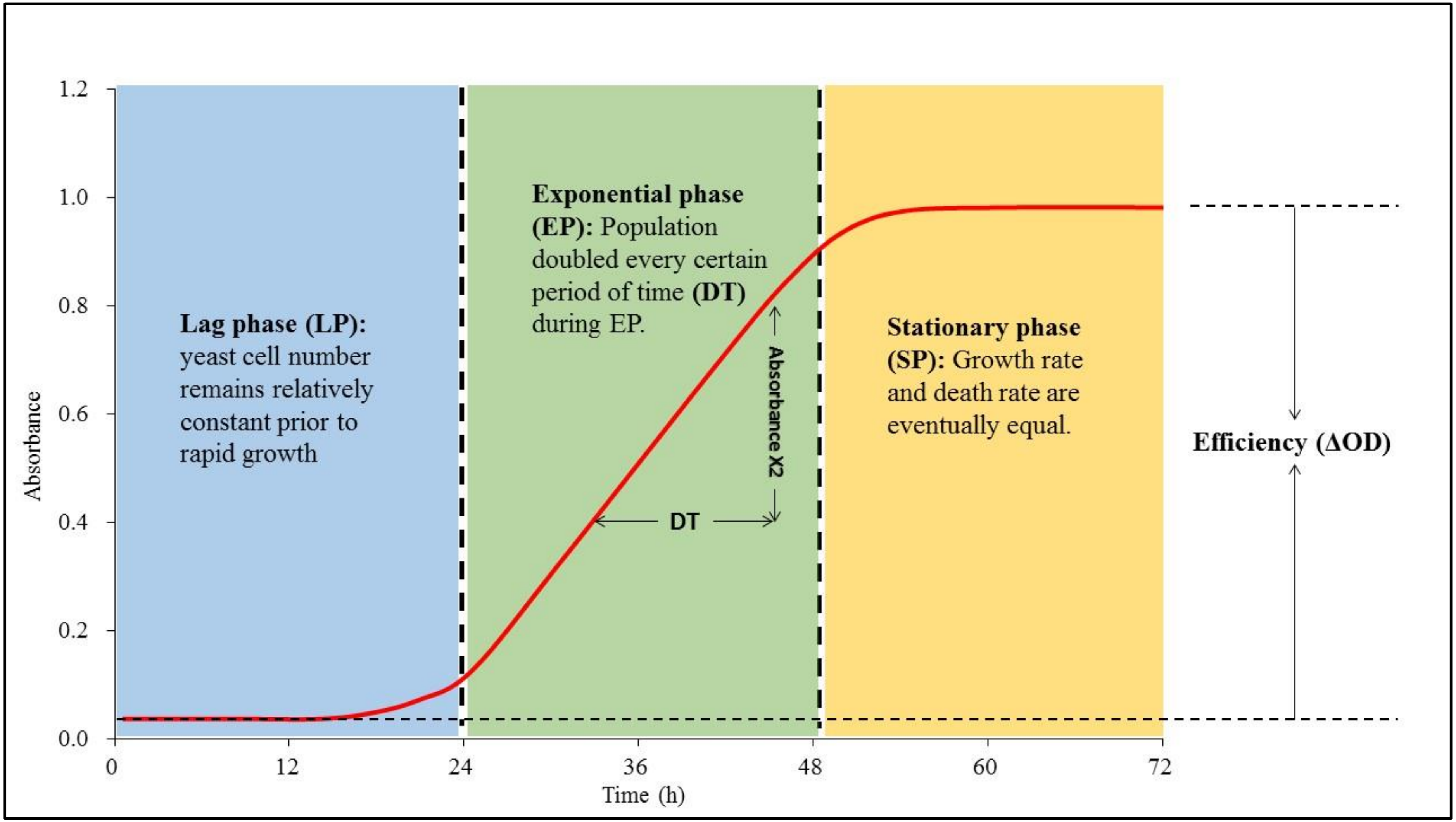

Supplement: Supplementary file 1 — Supplementary Information [file 41598_2017_14641_MOESM1_ESM.pdf]
